# Characterization and Regulation of Aquaporin Genes of Sorghum [*Sorghum bicolor* (L.) Moench] in Response to Waterlogging Stress

**DOI:** 10.3389/fpls.2017.00862

**Published:** 2017-05-30

**Authors:** Suhas Kadam, Alejandra Abril, Arun P. Dhanapal, Robert P. Koester, Wilfred Vermerris, Shibu Jose, Felix B. Fritschi

**Affiliations:** ^1^Division of Plant Sciences, University of Missouri, ColumbiaMO, United States; ^2^Graduate Program in Plant Molecular and Cellular Biology, University of Florida, GainesvilleFL, United States; ^3^Department of Microbiology and Cell Science – Institute of Food and Agricultural Sciences, University of Florida, GainesvilleFL, United States; ^4^University of Florida Genetics Institute, University of Florida, GainesvilleFL, United States; ^5^The Center for Agroforestry, University of Missouri, ColumbiaMO, United States

**Keywords:** sorghum, waterlogging, aquaporin, expression, haplotypes, phylogenetic

## Abstract

Waterlogging is a significant environmental constraint to crop production, and a better understanding of plant responses is critical for the improvement of crop tolerance to waterlogged soils. Aquaporins (AQPs) are a class of channel-forming proteins that play an important role in water transport in plants. This study aimed to examine the regulation of AQP genes under waterlogging stress and to characterize the genetic variability of AQP genes in sorghum (*Sorghum bicolor*). Transcriptional profiling of AQP genes in response to waterlogging stress in nodal root tips and nodal root basal regions of two tolerant and two sensitive sorghum genotypes at 18 and 96 h after waterlogging stress imposition revealed significant gene-specific pattern with regard to genotype, root tissue sample, and time point. For some tissue sample and time point combinations, *PIP2-6*, *PIP2-7*, *TIP2-2*, *TIP4-4*, and *TIP5-1* expression was differentially regulated in tolerant compared to sensitive genotypes. The differential response of these AQP genes suggests that they may play a tissue specific role in mitigating waterlogging stress. Genetic analysis of sorghum revealed that AQP genes were clustered into the same four subfamilies as in maize (*Zea mays*) and rice (*Oryza sativa*) and that residues determining the AQP channel specificity were largely conserved across species. Single nucleotide polymorphism (SNP) data from 50 sorghum accessions were used to build an AQP gene-based phylogeny of the haplotypes. Phylogenetic analysis based on single nucleotide polymorphisms of sorghum AQP genes placed the tolerant and sensitive genotypes used for the expression study in distinct groups. Expression analyses suggested that selected AQPs may play a pivotal role in sorghum tolerance to water logging stress. Further experimentation is needed to verify their role and to leverage phylogenetic analyses and AQP expression data to improve waterlogging tolerance in sorghum.

## Introduction

Aquaporins are integral membrane proteins that form channels that allow water to move from one plant compartment to another. They exist in all plants and animals and play important roles in different developmental and physiological processes of living organisms, including stomatal movement, photosynthesis, germination, cell elongation, reproduction, and responses to diverse abiotic stress conditions (Ariani and Gepts, 2015). In particular, AQPs play important roles in the regulation of plant water uptake, hydraulic conductivity, and water loss, and as such are critically involved in regulating tissue and whole-plant water relations (Chaumont and Tyerman, 2014). Other than water, AQPs can transport a variety of molecules including ammonia, CO_2_, boron, and silicon (Dordas et al., 2000; Terashima and Ono, 2002; Jahn et al., 2004; Ma et al., 2006). Plant AQPs were originally classified into four subfamilies: PIPs, TIPs, NIPs, and SIPs (Johanson et al., 2001; Zardoya, 2005). More recently, three additional AQP subfamilies, including glycerol facilitator (GlpF)-like intrinsic proteins (GIPs), hybrid intrinsic proteins (HIPs), and X (unrecognized) intrinsic proteins (XIPs) have been described. However, unlike PIPs, TIPs, NIPs, and SIPs which are present in all land plants, GIPs and HIPs have only been identified in algae and moss, and XIPs only in moss and several dicots (Danielson and Johanson, 2008; Venkatesh et al., 2013; Zhang et al., 2013). Two of the AQP subfamilies, the PIPs, which are usually localized in the plasma membrane, and the TIPs, which are generally localized in the vacuolar membranes, have been investigated intensively in regard to their functions and regulation as related to plant water relations.

Aquaporins have been studied extensively in order to understand the complex mechanisms of solute permeation and selectivity (Törnroth-Horsefield et al., 2005; Eriksson et al., 2013). AQPs are small proteins that are highly conserved in plants and animals and contain six transmembrane α-helix domains that form a pore. Two of the loops are characterized by NPA motifs (Asp-Pro-Ala) which, together with an aromatic/Arg filter (ar/R), act as a size-exclusion barrier and regulate the transport specificity of these proteins (Murata et al., 2000; Hub and De Groot, 2008; Mitani-Ueno et al., 2011).

As hypoxic or even anoxic conditions develop in the rhizosphere in response to waterlogging, growth of most plants is impaired. An early response to waterlogging is reduced water uptake by roots (Schildwacht, 1989; Else et al., 1995) caused by a reduction in root hydraulic conductance (Araki, 2006). A decrease in the root hydraulic conductance may result from a disruption of AQP function as a result of cytosol acidification (Tournaire-Roux et al., 2003) and may trigger water deficit stress leading to partial stomatal closure (Else et al., 2009). Additional responses to waterlogging include synthesis of the phytohormone ethylene, formation of aerenchyma in the root cortex facilitating oxygen diffusion, initiation and growth of adventitious roots, and development of radial oxygen loss barriers (Shaw, 2015). Numerous expression profiling studies have been conducted to elucidate molecular responses associated with low oxygen stress, including for Arabidopsis (Liu et al., 2005; Hsu et al., 2011; Van Veen et al., 2016), rice (*Oryza sativa* L.) (Lasanthi-Kudahettige et al., 2007), poplar (*Populus alba*) (Kreuzwieser and Gessler, 2010), sesame (*Sesamum indicum* L.) (Wang et al., 2012), and brassica (*Brassica napus* L.) (Zou et al., 2013). However, none of these studies focused on the impact of waterlogging on AQP transcript abundance in roots.

Many AQPs are known to be highly expressed in roots (Sakurai et al., 2005; Monneuse et al., 2011), supporting a role of AQPs in root water transport. Numerous recent studies have investigated the association between water relations and gene expression and/or protein levels of AQPs under various environmental conditions and in a range of plant species, providing information that may open the door to manipulating AQP expression to alter plant water-use efficiency (Moshelion et al., 2015). While AQP genes have been characterized in several plant species using genome-wide analyses (Matsuo et al., 2012; Deshmukh et al., 2015), information on AQPs in sorghum [*Sorghum bicolor* (L.) Moench] is sparse, particularly as related to waterlogging stress.

In low-laying areas along rivers in the United States Midwest, periodic short-term waterlogging is common and can cause significant biomass and yield losses. In the United States, losses in crop production due to flooding were second only to drought in many of the past years (Bailey-Serres et al., 2012). Waterlogging-prone land that is deemed too risky for the production of traditional row crops may be useful for the production of sorghum, a hardy C4 grass that originated in Africa. Sorghum is currently grown in the United States on >2.9 million ha^1^, mainly for the production of grain for use as animal feed, and, more recently, as bioenergy feedstock (Regassa and Wortmann, 2014). Cultivation of sorghum for the production of lignocellulosic biomass on waterlogging-prone land is of particular interest because this land is not used for the production of food crops, thus it would not redirect farmland normally used for food production for the production of biofuel (Leakey, 2009). This, coupled with only limited knowledge about sorghum responses to waterlogging (Zhang et al., 2016), highlights the need for research to elucidate the physiological and molecular responses of sorghum to waterlogging. Given the direct impact of waterlogging on plant roots, examination of root responses is of particular interest.

Sorghum’s seminal root system consists of the primary root and lateral branches that form on the primary root as the plant develops (Singh et al., 2010). In sorghum, nodal roots start to appear when plants have four to five fully expanded leaves. Nodal roots develop sequentially from shoot nodes in flushes that approximate the rate of new leaf appearance (Singh et al., 2010). As cereals develop from seedlings into mature plants, their nodal root systems develop into the dominant root system and provide most of the water and nutrients that are required (Krassovsky, 1926; Sallans, 1942; Shane and McCully, 1999). The limited information available in the literature indicates that continuous waterlogging of sorghum causes an increase in the number of nodal root axes, but not in their total length. Other root system components, such as nodal root laterals and the seminal root and its laterals, are restricted in number and length as a result of waterlogging (Pardales et al., 1991). Despite the availability of powerful genomic resources and techniques, the importance of AQPs in plant water relations, and the limited understanding of sorghum root responses to waterlogging, analyses of AQP gene expression in sorghum nodal roots are lacking.

Consequently, to better understand AQP gene expression in sorghum nodal roots in response to waterlogging stress we examined the transcript levels of selected sorghum AQP (*SbAQP*) genes in nodal root tips and root bases in genotypes contrasting in their response to waterlogging stress. Additionally, to gain a better understanding of SbAQPs and provide insights into the genetic variation as well as the association between single nucleotide polymorphism (SNP) haplotypes and waterlogging tolerance in sorghum, we established the phylogenetic relationship of sorghum AQPs with those of maize, rice, and Arabidopsis, assigned putative functions of SbAQPs, and performed haplotype analysis of AQP genes based on SNP data from 50 sorghum accessions.

## Materials and Methods

### Plant Culture and Waterlogging Stress Imposition

Mexico silt loam soil (fine, smectitic, mesic Aeric Vertic Epiaqualfs) was collected at the Bradford Research Center near Columbia, MO, United States. The soil obtained from the top 0.15 m of the profile was homogenized in a soil mixer and autoclaved before filling the pots (20 cm diameter; 32 cm tall). Three subsamples of soil were collected and submitted for analysis at the University of Missouri Soil and Plant Testing Laboratory. Test results indicated a salt pH of 6.5, 1.8% organic matter, 10 meq 100 g^-1^ cation exchange capacity, 58 kg P ha^-1^, 169 kg K ha^-1^, 3580 kg Ca ha^-1^, and 474 kg Mg ha^-1^. No fertilizer was applied during the experiment. Two waterlogging-tolerant and two waterlogging-sensitive sorghum genotypes from the ICRISAT mini-core collection (Upadhyaya et al., 2009) were selected for this study based on preliminary screening of the collection under waterlogging and control conditions in the field and follow-up characterization of selected entries under greenhouse conditions. Relative growth and leaf chlorosis of plants grown under waterlogged *versus* well-watered conditions were used as primary criteria to differentiate between waterlogging-tolerant and waterlogging-sensitive genotypes in the preliminary experiments (data not shown). Based on these experiments, genotypes IS 7131 and IS 10969 were characterized as tolerant and genotypes IS 12883 and IS 19389 were characterized as sensitive and were used for this study. Three seeds from each of these genotype were sown in 12 pots to accomodate four treatments and three replications. After sowing, one pot of each genotype was placed into a plastic tub (34 cm × 48 cm × 59 cm) to facilitate waterlogging treatment imposition. The resulting 12 tubs, each with one pot of each genotype, were arranged in three blocks of four tubs. After emergence, pots were thinned to one plant, and plants were watered regularly to maintain well-watered conditions until 30 days after sowing. At 30 days after sowing, plants had reached the V5 stage (Vanderlip, 1993) and waterlogging treatments were initiated by filling two tubs per replication with water, while maintaining the plants in the other two tubs well-watered. The water levels in the waterlogging treatments were maintained at 5 cm above the soil surface. Following the initiation of waterlogging, the redox potential at 5 cm soil depth was measured in each pot with a Pt electrode (HI3214P, Hanna instruments, Melrose, MA, United States) at 1, 18, and 96 h of waterlogging stress. At 18 h (short) and 96 h (long) post waterlogging treatment initiation, root tip and root base samples from control and waterlogged plants were harvested. To this end, plants were cut at the soil surface and roots were immediately removed from pots and washed by gentle agitation in a large, 30-L tub of water to remove all soil particles. Roots were quickly blotted dry with paper towels, weighed, and root tips (0 to 12 mm) and basal portions (20 mm region closest to the root-shoot junction) of second-whorl nodal roots were excised, immediately frozen in liquid N_2_, and stored at -80°C until RNA extraction. Care was taken to ensure that all root tip and basal tissue samples destined for quantification of transcript abundance were immersed in liquid N_2_ within 2 min following cutting of the shoot.

### RNA Isolation and qRT-PCR

A total of 96 root tissue samples were collected: four genotypes (two tolerant and two sensitive), two conditions (well-watered and waterlogged), two time points (18 and 96 h), two root regions (root tip and root base), and three biological replications. Tissue samples were ground with mortar and pestle in liquid N_2_, and RNA was extracted using the RNeasy Plant Mini kit (Qiagen, Germantown, MD, United States) according to manufacturer’s instructions. Extracted RNA was analyzed on a NanoDrop spectrophotometer (ND-1000, Thermo Scientific, Wilmington, DE, United States) to assess quantity and on a 1% (w/v) agarose gel to check quality. Template cDNA samples were prepared using Superscript II reverse transcriptase (Invitrogen, Carlson, CA, United States) with 500 ng of total RNA. Primers for reverse transcription PCR (RT-PCR) for nine *SbAQP* genes and actin were designed using Primer3 software^2^ to have a melting temperature between 58 and 62°C and to produce PCR products between 75 and 150 bp (Supplementary Table S1). The nine *SbAQP* genes included in this study were selected based on expression pattern revealed by RNAseq analysis of sorghum root tip and root base tissues in response to waterlogging (Supplementary Table S2, Kadam et al. unpublished results). The sorghum actin gene Sb01g0112600 was used to normalize gene expression, as its expression was found to be stable in root RNA extracted from different sorghum genotypes (Gelli et al., 2014). Transcript abundance was assayed using SYBR green PCR Master Mix (Applied Biosystems, Inc., Foster City, CA, United States) with 2 μl of 10-fold diluted cDNA and 1 μl of the primers (10 μM). The PCR program used was as follows: initial denaturation for 2 min at 95°C, followed by 40 PCR cycles consisting of 95°C for 15 s and 60°C for 60 s using an ABI 7500 thermal cycler (Applied Biosystems, United States). For each product, the threshold cycle (*C*_t_) where the amplification reaction enters the exponential phase, was determined for three independent biological replicates per genotype. The comparative *C*_t_ method was used to quantify the relative transcript abundance (Pfaffl, 2001). Gene expression data were analyzed by ANOVA using the GLM procedure in SAS 9.4 (SAS Institute, Cary, NC, United States). Mean separation was conducted by Tukey’s test at α = 0.05 and *n* = 3.

### Phylogenetic Analysis of AQP Proteins and Identification of NPA Motifs and ar/R Selectivity Filters

To identify putative AQP genes in sorghum, the protein sequences of all identified Arabidopsis, maize and rice AQPs were used as queries in BLASTX and BLASTP with default parameters from the NCBI and Phytozome databases. After filtering sorghum AQPs with at least 50% identity with the query sequence, the candidate AQP genes were aligned to ensure that no gene was represented multiple times. Multiple sequence alignments of AQPs identified by BLAST were performed using CLUSTALW implemented in MEGA7 (Kumar et al., 2016). The AQP alignments were used to construct a phylogenetic tree with the ML method using MEGA version 7. The stability of branch nodes in the ML-tree was measured by performing 1000 bootstraps and remaining parameters were kept at the default settings. The AQP subgroups PIP, TIP, NIP, and SIP formed in the phylogenetic tree were classified in accordance with the nomenclature of known AQPs, which were used as query in initial BLAST searches.

The conserved NPA domain and ar/R selectivity filter of the different subfamilies of sorghum AQPs were identified by multiple sequence alignment using the SeaView software (Gouy et al., 2010) with MUSCLE using default parameters.

### SNP Haplotype Analysis of *SbAPQ* Genes

A total of 50 sorghum accessions were used for SNP haplotype analysis. The SNP information of 48 sorghum accessions (46 *Sorghum bicolor* landraces, improved varieties, wild and weedy entries, and two *S. propinquum*) were obtained from a sorghum genome SNP database (Luo et al., 2016). In addition, the SNPs from the tolerant (IS 7131) and the sensitive (IS 12883) genotypes used in this study were obtained from RNAseq data (Kadam et al., unpublished). The SNP haplotype analysis was conducted using MEGA version 7 (Kumar et al., 2016) and complemented by manual analysis in Microsoft Excel. The ML method was used for the construction of trees and a bootstrap with 1,000 replicates was used to establish confidence in the branches.

## Results and Discussion

### Transcript Abundance of *SbAQPs* in Response to Waterlogging

Aquaporins play a major role in controlling hydraulic conductivity in leaves and roots (Chaumont and Tyerman, 2014; Sade et al., 2015). Consequently, the identification of physiologically important members and characterization of the regulation of their expression in response to waterlogging stress could be very helpful for crop improvement efforts. Analyses of RNAseq data (Supplementary Table S2, Kadam et al. unpublished) indicated that transcript abundance of the *SbAQP* genes encoding PIP1-6, PIP2-5, PIP2-6, PIP2-7, TIP2-1, TIP2-2, TIP4-4, TIP5-1, and NIP4-1 was influenced by waterlogging stress in sorghum roots. To further explore the expression of these genes, qRT-PCR analyses were conducted to quantify transcript abundance in root tips (0–12 mm region of the root apex) and root bases (20 mm region closest to the root–shoot junction) in two waterlogging-tolerant and two waterlogging-sensitive genotypes. Specifically, transcript abundance was examined in tissue samples collected from roots that developed from the second below-ground node in response to 18 and 96 h of waterlogging stress. The root tip samples were collected to represent the growing region of the second nodal root, thus including the root apical meristem as well as cells that are expanding. In contrast, the samples collected from the base of the root consist of mature cells. As such, the two regions represent tissues that differ substantially in anatomy, physiology, and biochemistry, as well as in their response to waterlogging stress (Xu et al., 2013).

**FIGURE 1:**
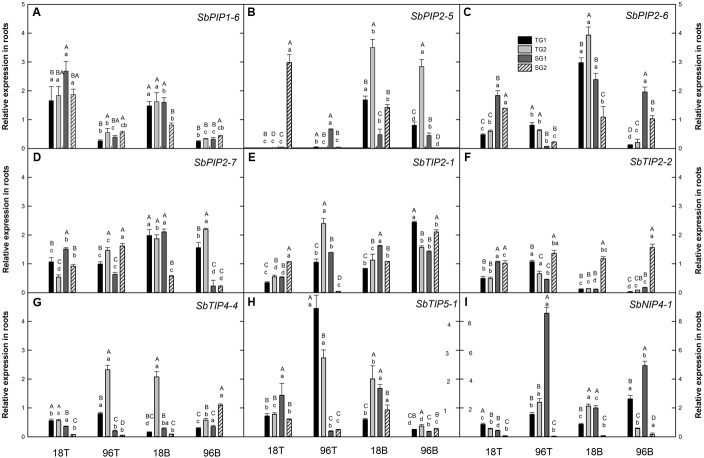
**Relative expression of aquaporin genes in sorghum root tip (T) and root base (B) tissues after 18 and 96 h of waterlogging stress** **(A)** *PIP1-6* (Sobic.010G087900) **(B)** *PIP2-5* (Sobic.006G150100) **(C)** *PIP2-6* (Sobic.002G125000) **(D)** *PIP2-7* (Sobic.002G281000) **(E)** *TIP2-1* (Sobic.004G295100) **(F)** *TIP2-2* (Sobic.010G146100) **(G)** *TIP4-4* (Sobic.003G007200) **(H)** *TIP5-1* (Sobic.006G170500) **(I)** *NIP4-1* (Sobic.003G098100). Different letters indicate significant differences (*P* < 0.05) between genotypes (uppercase letters) within tissue and time point, and between tissues and time points for a particular genotype (lowercase letters). Error bars indicate standard error (*n* = 3). TG1, tolerant genotype IS 7131; TG2, tolerant genotype IS 10969; SG1, sensitive genotype IS 12883; SG2, sensitive genotype IS 19389; T, root tip; B, root base.

Analysis of variance indicated significant differences in transcript abundance between control and waterlogged treatments for all nine *SbAQP* genes in tolerant as well as in sensitive genotypes and in root tips as well as root bases. However, the responses to waterlogging stress differed depending on *SbAQP* gene, genotype, tissue type, and waterlogging stress duration (**Figure 1** and Supplementary Table S3). Further, strong effects of waterlogging stress duration (18h vs. 96) on transcript abundance were observed, for most *SbAQP* genes regardless of tissue type and genotype. Similarly, waterlogging treatment effects on transcript abundance was often different between root tip and root base samples. These results are consistent with measurements of *AQP* transcript abundance in response to waterlogging stress in two *Quercus* species in that distance of the collected sample from the root apex, stress duration, and species all influenced transcript levels of different *AQPs* (Rasheed-Depardieu et al., 2015).

In the present study, among all *SbAQP* genes examined, the expression pattern of *SbPIP1-6* was the most consistent among genotypes. It was the only gene which was upregulated in both root tip and root base samples at 18 h and downregulated at 96 h in all genotypes (except IS 19389 at 18 h in the root base) (**Figure 1A**). Previously, expression of *SbPIP1-6* in sorghum leaves was found to be downregulated as a result of 4 h cold and heat stress, 24 h salt stress and 5 days drought stress (Reddy et al., 2015). In roots, the observed initial upregulation of *SbPIP1-6* expression in response to short-term exposure to stress may enhance water uptake to maintain the plant water status, while reduced expression after prolonged exposure to stress may reduce hydraulic conductivity. Based on the MOROKOSHI sorghum transcriptome database (Makita et al., 2014), the expression of *SbPIP1-6* in roots is responsive to a range of treatments including nitrogen, polyethylene glycol, abscisic acid, and NaOH. Together, these results indicate that consistent with an important role of SbPIP1-6, expression of *SbPIP1-6* in roots is responsive to changes in a broad range of environmental conditions, but the expression pattern observed in this study does not indicate an association between *SbPIP1-6* transcript abundance and the waterlogging tolerance or sensitivity of the four sorghum genotypes.

While differences among the four genotypes were common, transcript abundance of specific *SbAQP* genes often did not display consistent contrasts between the tolerant and the sensitive genotypes. That said, instances of expression pattern that were associated with tolerance/sensitivity of the genotypes were found for *SbPIP2-6*, *SbPIP2-7*, *SbTIP2-2*, *SbTIP4-4*, and *SbTIP5-1*. In particular, transcript abundance of *SbPIP2-6* in root tips was significantly different between the tolerant and the sensitive genotypes at both 18 and 96 h (**Figure 1C**). At 18 h, *SbPIP2-6* expression was upregulated in the sensitive genotypes but not in the tolerant genotypes, while at 96 h it was downregulated in all genotypes but to a greater extent in the sensitive genotypes than the tolerant genotypes. Interestingly, *SbPIP2-6* expression was also upregulated in the sensitive and downregulated in the tolerant genotypes in the root bases in response to prolonged stress, while its expression was upregulated in the root bases of all genotypes at 18 h. Aside from *SbPIP2-6*, consistent significant differences in transcript abundance between sensitive and tolerant genotypes at more than one time point within the same tissue were only found for *SbTIP4-4* (**Figure 1G**). The expression of *SbTIP4-4* was downregulated in both sensitive genotypes in root tips collected at 18 and 96 h of waterlogging, and the transcript abundance was significantly lower than in the two tolerant genotypes. In the case of *SbPIP2-7*, *SbTIP2-2*, and *SbTIP5-1*, one time point and tissue combination each was found for which the sensitive and tolerant genotypes exhibited consistent expression patterns. In response to prolonged waterlogging stress, expression of *SbPIP2-7* and *SbTIP5-1*, were upregulated in the tolerant and downregulated in the sensitive genotypes in root bases and root tips, respectively (**Figures 1D,H**). In the root base at 96 h, *SbTIP5-1* was not only downregulated in the sensitive genotypes but also in the tolerant genotypes. Tolerant genotypes downregulated the expression of *SbTIP2-2* in root tips in response to 18 h of waterlogging stress while the transcript abundance in sensitive genotypes changed little compared to the control treatment (**Figure 1F**). The expression of *SbTIP2-2* in the root bases of three of the four genotypes was strongly downregulated in response to 18 h as well as 96 h of waterlogging, and the changes in transcript abundance in response to waterlogging stress were much greater than in the root tips. Downregulation of expression in the root base may be correlated with aerenchyma formation due to cortical cells death in waterlogging stress. A greater degree of root aerenchyma formation in the root base has been reported for maize and sorghum compared to root tips in response to waterlogging stress (Mano et al., 2006; Promkhambut et al., 2011). While significant differences between genotypes and/or time points were observed for transcript abundance of *SbTIP2-1* and *SbNIP4-1*, the only consistent response to waterlogging that was observed for these genes was that *SbNIP4-1* expression in one of the susceptible genotypes (IS 19389) was strongly downregulated in both tissues at both time points (**Figure 1I**). Given the recent finding SbNIP4-2 can transport silicon (Markovich et al., 2015), it is possible that the distinct expression pattern of SbNIP4-1 in IS 19389 (SG2) may also result in silicon accumulation differences in comparison with the other three genotypes. Interestingly, in flood-stressed Arabidopsis, NIP2-1 was induced and may play a role in adaptation to lactic fermentation (Choi and Roberts, 2007).

Diverse patterns in transcript abundance such as those documented here for different AQP genes are not surprising and consistent with AQP expression responses to abiotic stresses, including waterlogging stress, that have been observed by others (Weig et al., 1997; Mariaux et al., 1998; Jang et al., 2004; Ge et al., 2014; Rasheed-Depardieu et al., 2015), as well as differences in cellular location and transport functions that have been documented for AQPs (Hu et al., 2015; Reddy et al., 2015; Deshmukh et al., 2016). Additionally, given the distinct developmental age and associated physiology of the root tip vs. root base tissues, distinct expression pattern and transcript abundance among *SbAQP* genes between the tissues could be expected and have also been observed by others (Rasheed-Depardieu et al., 2015).

Differences in gene expression between tolerant and sensitive genotypes may or may not be linked to their performance under waterlogged conditions. Nonetheless, here, genes for which the expression pattern of the two tolerant genotypes were similar and different from the two sensitive genotypes were regarded as more likely to be functionally associated with sensitivity or susceptibility to waterlogging stress. Such expression patterns were observed for *SbPIP2-6, SbTIP2-2*, *SbTIP4-4*, and *SbTIP5-1* in root tips and for *SbPIP2-6* and *SbPIP2-7* in root bases (**Figure 1**). Recently, Sutka et al. (2011) reported that the transcript abundance of several *PIPs* (*AtPIP1;1, AtPIP1;2, AtPIP1;4, AtPIP2;1, AtPIP2;3, AtPIP2;4* and *AtPIP2;5)* in Arabidopsis roots is positively correlated with hydraulic conductivity; and, increased expression levels may regulate the uptake of water into cells (Suga et al., 2002). As such, the above-described contrasts in *SbPIP2-6* and *SbPIP2-7* transcript abundance between sensitive and tolerant genotypes may be associated with differences in hydraulic conductivity. Interestingly, opposing expression pattern were observed for these two members of the *SbPIP* family in the root base tissues of susceptible and tolerant genotypes at 96 h, in that *SbPIP2-7* was upregulated in the tolerant but downregulated in the susceptible genotypes while *SbPIP2-6* was downregulated in the tolerant but upregulated in the susceptible genotypes. In any case, the relevance of these genes and the associated expression differences with regard to waterlogging tolerance/sensitivity as well as hydraulic conductivity remain to be examined.

Three members of the *SbTIP* family, namely *SbTIP2-2*, *SbTIP4-4*, and *SbTIP5-1*, exhibited expression pattern differences between sensitive and tolerant genotypes in root types at 18 and/or 96 h (**Figures 1F,G,H**). In Arabidopsis, AtTIP facilitates the transport of water, hydrogen peroxide, and urea (Bienert et al., 2007), and SbTIPs likely have similar functions in sorghum. It is interesting to speculate whether the observed gene expression responses primarily influence water transport or whether they may play an important role relative to hydrogen peroxide transport under waterlogged conditions. Hydrogen peroxide is known to be involved in the regulation of root growth, probably acting downstream of auxin (Ivanchenko et al., 2013), and is produced in cortical cells in wheat seminal roots undergoing aerenchyma formation in response to waterlogging (Xu et al., 2013). Thus, different expression pattern of *SbTIP2-2*, *SbTIP4-4*, and *SbTIP5-1* in root tips of sensitive and tolerant genotypes may result in contrasting hydraulic conductivity and/or may alter the distribution of hydrogen peroxide in root tip cells. In root base tissues, no consistent differences in the expression of *SbTIPs* were found between sensitive and tolerant genotypes, but *SbTIP2-1* was upregulated in three of the four genotypes at 18 h as well as 96 h of waterlogging stress (**Figure 1E**). Further research is needed to examine whether the greater abundance of *SbTIP2-1* transcripts in the root base tissue is associated with hydrogen peroxide formation and the development of aerenchyma in these samples.

The impact of waterlogging stress on transcript abundance in nodal roots was dependent on the position of the root tissue sample, duration of stress imposition, and the sorghum genotype, and differed among the examined *SbAQP* genes. Although some consistent responses across the sensitive and the tolerant genotypes were detected for some of the genes, tissues, and/or time points, it remains unclear whether any of the observed responses reflect differences in AQP abundance and/or are functionally related to the tolerance/sensitivity of the genotypes.

### Phylogenetic Analysis of the AQP Family

**FIGURE 2:**
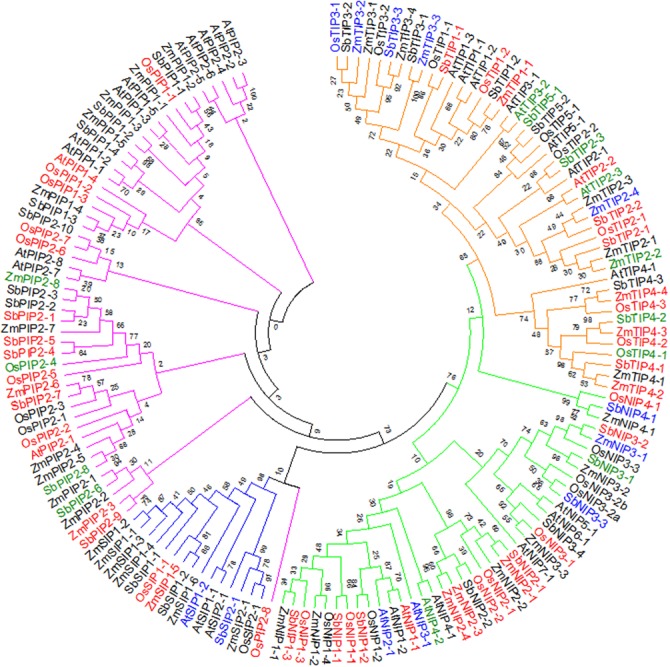
**Phylogenetic analysis showing relative closeness of sorghum AQP proteins with maize rice and Arabidopsis AQP proteins.** The tree was constructed using the maximum likelihood method with bootstrap from full-length amino acid sequences of maize, rice, Arabidopsis, and sorghum AQP proteins. The color bar represents the subfamily of AQPs (Red – TIP, Green – NIP, Blue – SIP, Purple – PIP). Aquaporins highlighted in red, blue, or green font indicate genes for which differences transcript abundance in response to waterlogging-related stresses was documented in this or previous studies of Arabidopsis (Liu et al., 2005; Mustroph et al., 2014; Van Veen et al., 2016), rice (Lasanthi-Kudahettige et al., 2007; Narsai et al., 2011; Kawahara et al., 2016), and maize (Campbell et al., 2015) (red: downregulated; blue: upregulated; green: up- or down-regulated).

The differential regulation of the AQP genes as established above, raises questions about how sorghum AQP genes relate to those of other plant species. Advances in plant genome sequencing have enabled the identification and characterization of AQPs in several crop species including rice, maize, and soybean (*Glycine max* L.) (Chaumont et al., 2001; Sakurai et al., 2005; Zhang et al., 2013), facilitating comparative analyses with sorghum AQPs. To study the relationships among AQP proteins from sorghum, maize, rice, and Arabidopsis, a phylogenetic tree was created based on amino acid sequence alignments (**Figure 2**). Consistent with previous reports for AQPs in rice, Arabidopsis, and maize, sorghum AQPs grouped into PIP, TIP, NIP, and SIP subfamilies (Weig et al., 1997; Chaumont et al., 2001; Sakurai et al., 2005). The SbPIP subfamily was the largest, with 14 members divided into two groups: PIP1, with 4 members and PIP2, with 10 members. Five groups were found for the SbTIP subfamily (TIP1 to TIP5), with two members in the TIP1 group, three each in groups TIP2 to TIP4 and two members in the TIP5 group. Sorghum NIPs were divided into NIP1 with three members, NIP2 with two members, NIP3 with four members, and NIP4 with one member. With only three members, the SbSIP subfamily was the smallest and had two SIP1 members and one SIP2 member (**Figure 2**). Among the four species compared in this study, the number of PIPs was greatest in sorghum and the number of SIPs was greatest in maize. Examination of protein sequences revealed higher levels of similarity of SbPIP members with maize, rice, and Arabidopsis PIP families, than between SbTIP, SbNIP, and SbSIP members and their respective families in maize, rice, and Arabidopsis. Information on differential expression of AQP genes response to waterlogging, submergence and hypoxic stress obtained from the public domain (Supplementary Table S4) (Liu et al., 2005; Lasanthi-Kudahettige et al., 2007; Narsai et al., 2011; Mustroph et al., 2014; Campbell et al., 2015; Kawahara et al., 2016; Van Veen et al., 2016)^3^, indicates that waterlogging influences transcript abundance of many AQPs (**Figure 2**). As in the present study, it appears that the transcript abundance of most AQP genes is lower in roots from waterlogged compared to control treatments.

### Analysis of Conserved and Substrate Specific Residues in AQP Proteins

The two NPA motifs found in AQPs are critical for water transport and selectivity (Murata et al., 2000). In addition, the ar/R selectivity filter is essential in determining transport specificity of AQPs. Point mutations or other sequence variations in these residues confer different substrate specificities to AQPs (Beitz et al., 2006; Hub and De Groot, 2008; Mitani-Ueno et al., 2011). To understand the possible physiological role and substrate specificity of sorghum AQPs, we identified and examined the NPA motifs and ar/R selectivity filter sequences (**Table 1**). The two NPA domains were conserved in the SbPIP and SbTIP AQP subfamilies, except for SbTIP5-2. In SbTIP5-2, the asparagine was replaced by a threonine residue in the first NPA domain (NPA to TPA) and the second NPA domain was replaced by HEP (His-Glu-Pro) (**Table 1**). Interestingly, this change in both NPA domains of SbTIP5-2 was not observed in maize, rice, and Arabidopsis (Supplementary Table S5).

**Table 1:** Details of NPA domains, ar/R filters and Froger’s residues identified using protein sequence alignment in 40 sorghum aquaporins.

S. No.	Gene_Id	NPA (I)	NPA (II)	ar/R filters				Froger’s residues				
					
				H2	H5	LE1	LE2	P1	P2	P3	P4	P5
1	*SbPIP1-1*	NPA	NPA	F	H	T	R	Q	S	A	F	W
2	*SbPIP1-2*	NPA	NPA	F	H	T	R	Q	S	A	F	W
3	*SbPIP1-3*	NPA	NPA	F	H	T	R	Q	S	A	F	W
4	*SbPIP1-4*	NPA	NPA	F	H	T	R	V	S	A	F	W
5	*SbPIP2-1*	NPA	NPA	F	H	T	R	Q	S	A	F	W
6	*SbPIP2-2*	NPA	NPA	F	H	T	R	Q	S	A	F	W
7	*SbPIP2-3*	NPA	NPA	F	H	T	R	Q	S	A	F	W
8	*SbPIP2-4*	NPA	NPA	F	H	T	R	Q	S	A	F	W
9	*SbPIP2-5*	NPA	NPA	-	H	T	R	Q	S	A	F	W
10	*SbPIP2-6*	NPA	NPA	F	H	T	R	Q	S	A	F	W
11	*SbPIP2-7*	NPA	NPA	F	H	T	R	Q	S	A	F	W
12	*SbPIP2-8*	NPA	NPA	F	H	T	R	Q	S	A	F	W
13	*SbPIP2-9*	NPA	NPA	F	H	T	R	H	S	A	F	W
14	*SbPIP2-10*	NPA	NPA	F	H	T	R	M	S	A	F	W
15	*SbTIP1-1*	NPA	NPA	H	I	A	V	T	S	A	Y	W
16	*SbTIP1-2*	NPA	NPA	H	I	A	V	T	S	A	Y	W
17	*SbTIP2-1*	NPA	NPA	R	I	G	R	T	S	A	Y	W
18	*SbTIP2-2*	NPA	NPA	H	I	G	R	T	S	A	Y	W
19	*SbTIP2-3*	NPA	NPA	H	I	G	R	T	S	A	Y	W
20	*SbTIP3-1*	NPA	NPA	H	V	A	R	T	A	A	Y	W
21	*SbTIP3-2*	NPA	NPA	H	V	A	R	T	V	A	Y	W
22	*SbTIP3-3*	NPA	NPA	H	I	A	R	S	A	A	Y	W
23	*SbTIP4-1*	NPA	NPA	H	S	A	R	S	S	A	Y	W
24	*SbTIP4-2*	NPA	NPA	Q	S	A	R	T	S	A	Y	W
25	*SbTIP4-3*	NPA	NPA	H	V	A	R	T	S	A	Y	W
26	*SbTIP5-1*	NPA	NPA	Q	V	A	R	S	S	A	Y	W
27	*SbTIP5-2*	TPA	HEP	Q	V	G	G	S	–	A	Y	W
28	*SbNIP1-1*	NPA	NPA	W	V	A	R	F	S	A	Y	V
29	*SbNIP1-2*	NPA	NPA	W	V	A	R	F	T	A	Y	M
30	*SbNIP1-3*	NPA	NPA	W	V	A	R	F	T	A	Y	F
31	*SbNIP2-1*	NPA	NPA	G	S	G	R	L	T	A	Y	F
32	*SbNIP2-2*	NPA	NPA	G	S	G	R	L	T	A	Y	F
33	*SbNIP3-1*	NPA	NPA	A	A	A	R	Y	T	A	Y	V
34	*SbNIP3-2*	NPA	NPA	A	–	P	R	Y	T	A	Y	L
35	*SbNIP3-3*	NPA	NPA	A	A	–	R	Y	T	A	Y	M
36	*SbNIP3-4*	NPS	NPV	A	I	G	R	F	T	A	Y	L
37	*SbNIP4-1*	NPA	NPI	C	G	G	R	M	T	A	Y	L
38	*SbSIP1-1*	NPT	NPA	L	I	P	N	S	A	A	Y	W
39	*SbSIP1-2*	NPT	NPA	L	V	P	N	S	A	A	Y	W
40	*SbSIP2-1*	NPL	NPA	S	H	G	S	V	A	A	Y	W


Except for SbNIP3-4 and SbNIP4-1, both NPA domains were conserved in all members of the SbNIP subfamily. In SbNIP3-4, the alanine in the first NPA domain was substituted with a serine, and in the second NPA domain it was substituted with a valine. While the first NPA domain was conserved in SbNIP4-1, the alanine was substituted with isoleucine in the second domain (**Table 1**). AQPs in the SbSIP subfamily had a conserved NPA motif in the second domain, but the alanine in the first domain was replaced with either a threonine (SbSIP1-1 and SbSIP1-2) or a leucine (SbSIP2-1) (**Table 1**). Further research is needed to understand the implications associated with the modified NPA domains in SbTIP5-2, SbNIP3-4, SbNIP4-1, and the SbSIPs.

All but one (PIP2-5) identified SbPIPs contained the ar/R selectivity filter that is highly conserved and typical of water-transporting AQPs (F/H/T/R) (**Table 1**). The same ar/R selectivity filter sequence is shared by the PIP subfamily in different plant species including maize, Arabidopsis, white poplar, tomato (*Solanum lycopersicum*), soybean, and canola (*Brassica rapa*) (Chaumont et al., 2001; Johanson et al., 2001; Gupta and Sankararamakrishnan, 2009; Reuscher et al., 2013; Zhang et al., 2013; Tao et al., 2014). Evidence is mounting that PIP AQPs are actively involved in regulating root and leaf hydraulic conductivity (Javot et al., 2003; Sutka et al., 2011; Sade et al., 2014; Grondin et al., 2016; Vitali et al., 2016). In addition to water transport, PIP AQPs in Arabidopsis, tobacco (*Nicotiana tabacum*), and barley (*Hordeum vulgare*) have been shown to facilitate diffusion of CO_2_ in leaf mesophyll cells and can directly affect photosynthesis (Flexas et al., 2006; Heckwolf et al., 2011). The conservation of NPA motifs and ar/R residues in sorghum PIPs suggests that they are involved in regulating water absorption, plant hydraulics, and/or CO_2_ diffusion (Reddy et al., 2015).

Aquaporins of the TIP subfamily are found mostly in vacuolar membranes and are involved in the control of osmotic potential and water flow across this plant subcellular compartment (Maurel et al., 1993; Pou et al., 2013). A number of studies revealed that TIPs can transport a variety of small solutes, such as NH_4_^+^, hydrogen peroxide, and urea, in addition to water (Liu et al., 2003; Holm et al., 2005; Loqué et al., 2005; Bienert et al., 2007). The conserved ar/R residues and NPA motifs of SbTIPs compared with those of other species, suggest a conserved function for these proteins in sorghum (Supplementary Table S5). Waterlogged conditions have been shown to promote the production of reactive oxygen species including hydrogen peroxide in roots and leaves of barley (Kalashnikov et al., 1994) and roots of wheat (Biemelt et al., 2000). Consequently, differences in transcript abundance of *SbTIP2-2*, *SbTIP4-4*, and *SbTIP5-1* between tolerant and sensitive sorghum genotypes (**Figure 1**) may point to distinct hydrogen peroxide distribution and/or production in roots of these genotypes.

For the SbNIPs, six distinct ar/R selectivity filters were identified. All three SbNIP1 members had the residues W/V/A/R typical of the subgroup I of plant NIP AQPs (**Table 1** and Supplementary Table S4). These NIPs are able to transport uncharged solutes like glycerol and formamide but have low water permeability (Wallace and Roberts, 2005). The structural similarities of the SbNIP1 group with those of other plant species suggest analogous transport specificity. Rice NIP2-1 is a silicon transporter characterized by a double NPA motif and a G/S/G/R ar/R selectivity filter (Ma et al., 2006), and is able to transport arsenite and boric acid when expressed in Xenopus oocytes (Mitani-Ueno et al., 2011). Since the SbNIP2-1 sequence is similar to that of OsNIP2-1, it may be involved in both silicon and boric acid homeostasis in sorghum (**Table 1**). The Arabidopsis boric acid transporter NIP5-1 is characterized by an NPS/NPV aqueous pore and an A/I/G/R selectivity filter (Takano et al., 2006), and modifications in the protein sequence alter the transport capability of this AQP. Given that AtNIP5-1 and its sorghum homolog SbNIP3-4, share the same domain and selectivity filter, SbNIP3-4 may be involved in boron transport in sorghum. Comparison of NPA and ar/R motifs between the tolerant (IS 7131) and sensitive (IS 12883) sorghum genotypes, did not reveal any non-synonymous SNPs in the NPA and ar/R motifs (Supplementary Table S6).

### Haplotype Analysis of *SbAQP* Genes

**FIGURE 3:**
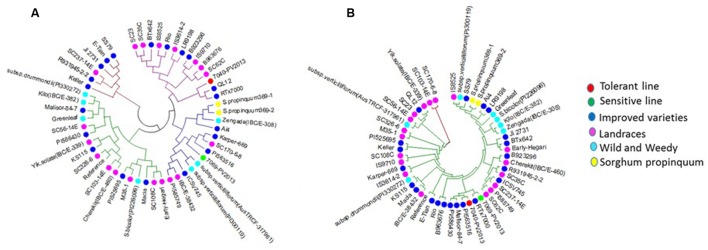
**Cluster analysis of** **(A)** *PIP1-6* (10G087900) and **(B)** *PIP2-7* (02G281000). The colors of the circles represent the different groups of sorghum genotypes (Red – tolerant genotype, Green – sensitive genotype, Blue – improved varieties, Purple – landraces, Light blue – wild and weedy, and Yellow – *S. propinquum*. Tolerant genotype (TG1) IS 7131 (7040-PV2013), sensitive genotype (SG1) IS 12883 (7069-PV2013).

Reduced cost of next-generation sequencing has opened the door for the generation of high-density SNP information of sorghum accessions (Morris et al., 2013; Luo et al., 2016), and for the detection of rare alleles which can be used to differentiate sorghum accessions. Haplotype analysis of *SbAQP* genes was accomplished using 50 sorghum accessions. The SNP information of 48 accessions was obtained from the sorghum SNP database (Luo et al., 2016) and the SNP information for the remaining two accessions was based on sequence information resulting from the RNAseq analysis of a waterlogging-tolerant (IS 7131) and a waterlogging-sensitive (IS 10969) genotype (Kadam et al. unpublished data). Sequence comparison between the waterlogging tolerant and sensitive genotype identified SNPs in six out of nine selected *SbAQP* genes [Sobic.002G125000 (*PIP2-6*), Sobic.002G281000 (*PIP2-7*), Sobic.003G098100 (*NIP4-1*), Sobic.006G170500 (*TIP5-1*), Sobic.010G146100 (*TIP2-2*), and Sobic.010G087900 (*PIP1-6*)]. No SNPs were identified for Sobic.06G150100 (*PIP2-5*), Sobic.003G007200 (*TIP4-4*), and Sobic.04G295100 (*TIP2-1*) (Supplementary Table S6). Most of the SNPs were present in the 3′ and 5′ UTR. The haplotype range was 5 to 35 (**Table 2**), with the fewest haplotypes present in *TIP4-4* (Sobic.003G007200) and the most in *NIP4-1* (Sobic.003G098100) (**Table 2**). For *SbPIP1-6* (Sobic.010G087900.1) a total of 27 haplotypes were present among the 50 sorghum accessions, and in the cluster analysis the tolerant (IS 7131) and sensitive (IS 12883) genotypes were assigned into separate groups (**Figure 3** and Supplementary Table S6). Interestingly, in root tips sampled 18 h into the waterlogging treatment, transcript levels of *SbPIP1-6* were significantly higher in IS 12883 (sensitive; SG1) than IS 7131 (tolerant; TG1). Whether the majority of the 50 sorghum accessions which have the same haplotype as the waterlogging-tolerant genotype also exhibit similar expression pattern for *PIP1-6* as IS 7131, remains to be examined. For *SbPIP2-7*, a total of 16 haplotypes were present, and, although the tolerant and sensitive genotypes had different haplotypes (Supplementary Table S6), they were assigned to the same group based on the cluster analysis. While no consistent expression patterns were observed for sensitive and tolerant genotypes in root tips and in the root base at 18 h, *SbPIP2-7* expression was upregulated in the tolerant and downregulated in the sensitive genotypes in the basal root region at 96 h of waterlogging (**Figure 1D**). Only one SNP was present in the 3′ UTR of *SbPIP2-7* between the sensitive and tolerant genotypes. Additional studies are needed to determine whether this SNP is causing the differential expression observed in the basal root region at 96 h. Seven *SbTIP5-1* haplotypes were identified among the 50 examined accessions (Supplementary Table S6). The tolerant and sensitive genotypes differed by one nucleotide in the 3′ UTR of *SbTIP5-1* which may underlie differential expression of this gene between these genotypes. In the tolerant genotype, *SbTIP5-1* expression increased in root tips with prolonged (96 h) exposure to waterlogging (**Figure 1E**). In contrast, in root tips of the sensitive genotype, *SbTIP5-1* transcript abundance was greater at 18 h compared to 96 h. Increased expression of this gene has also been reported in leaves in response to drought stress in sorghum and banana (*Musa acuminata* L.) (Hu et al., 2015; Reddy et al., 2015). While four haplotypes were observed for *SbTIP4-4*, no sequence differences were present between the tolerant and sensitive genotype for this gene, and thus, differences in transcript abundance for this gene were not associated with differences in its sequence (**Figure 1G** and Supplementary Table S6). Analysis of *SbTIP2-2* sequences revealed 29 haplotypes as well as differences in three nucleotides between the tolerant and sensitive genotype. Despite these three differences, the two genotypes were assigned to the same cluster.

**Table 2:** Number of SNPs in selected nine AQP genes with their distribution in non-synonyms, synonyms, 3′ UTR, and 5′ UTR regions of the genes.

Sorghum gene ID	AQPs	Haplotypes	Total SNPs	3′ UTR	Non-syn	Syn	5′ UTR	Start lost or splice region variant
Sobic.002G125000	*PIP2-6*	11	45	25	3	6	11	0
Sobic.002G281000	*PIP2-7*	16	20	9	2	2	6	1
Sobic.003G007200	*TIP4-4*	5	5	1	1	2	1	0
Sobic.003G098100	*NIP4-1*	35	74	7	7	13	36	11
Sobic.004G295100	*TIP2-1*	6	18	6	1	6	5	0
Sobic.006G150100	*PIP2-5*	16	50	9	0	3	38	0
Sobic.006G170500	*TIP5-1*	6	12	5	1	2	4	0
Sobic.010G087900	*PIP1-6*	27	23	13	4	3	3	0
Sobic.010G146100	*TIP2-2*	29	30	17	0	6	7	0


*Haplotype number represents analysis based on 50 sorghum accessions.*

Different expression pattern of some of the AQP genes in tolerant as compared to sensitive genotypes under waterlogging stress, and SNPs that may underlie these expression differences may be associated with the tolerance phenotype, and, if so, could prove useful for the development of molecular markers to screen populations of sorghum for waterlogging tolerance. Phylogenetic analysis on the basis of gene-based haplotypes is useful for the selection of tolerant genotypes (Kadam et al., 2016) because characterization of plant sensitivity or tolerance to waterlogging stress is time-consuming and costly. However, additional research is needed to determine the relevance of SNPs in AQP genes relative to waterlogging tolerance and could be coupled with efforts to identify genetic markers for waterlogging tolerance using bi-parental populations or diversity panels.

## Conclusion

In this study, 40 AQP genes were identified in the *Sorghum bicolor* genome and were phylogenetically grouped into four subfamilies. Phylogenetic comparisons of rice, maize, Arabidopsis, and sorghum AQP proteins showed that homologous pairs were clustered together into a single class. Expression profiling of AQP genes revealed differences in transcript abundance between plants subjected to waterlogging and well-watered control plants in a tissue-type and sampling-time dependent manner. Further, the expression pattern of specific AQP genes often differed based on genotype, independent of the genotype’s sensitivity to waterlogging. However, transcript abundance of *PIP2-6*, *PIP2-7*, *TIP2-2*, *TIP4-4*, and *TIP5-1* exhibited contrasting pattern in tolerant and sensitive genotypes for some tissue-type and sampling-time combinations, and thus may in part contribute to the differences in performance of these genotypes under waterlogged conditions. SNP identification and haplotype analysis within a diverse set of sorghum genotypes identified genic variation in AQP genes, which may be useful in sorghum breeding efforts. Further studies are required to ascertain the relevance and specific functions of the different genes in terms of waterlogging stress tolerance.

## Author Contributions

SK: Designed the study, performed data analysis, and wrote the manuscript; AD: participated in the data analysis; RK and AA: participated in experiment setup and measurements; WV and SJ: participated in the design and editing of the manuscript; FF: designed and supervised the research and contributed to the writing of the manuscript. All the authors read and approved the final version of the manuscript.

## Conflict of Interest Statement

The authors declare that the research was conducted in the absence of any commercial or financial relationships that could be construed as a potential conflict of interest.

## Footnotes

1 http://www.nass.usda.gov

2 http://frodo.wi.mit.edu

3 http://tenor.dna.affrc.go.jp/

## Abbreviations

AQP: aquaporin
ML: maximum likelihood
NIP: NOD26-like intrinsic protein
PIP: plasma membrane intrinsic protein
qRT-PCR: quantitative real time PCR
SIP: small basic protein
TIP: tonoplast intrinsic protein
